# The Post-War Probationer

**Published:** 1923-06

**Authors:** 


### The Post-War Probationer.

SIR,—In your note on "The Post-War Probationer" in THE HOSPITAL AND HEALTH REVIEW for May, you ask for your readers' views on the problem of the dearth of probationers.

We are two nurses now in training, and would like to state our views. We know that nursing must necessarily be a very strenuous life, but surely the work could be reduced from a 10-hour day (totalling 60 hours a week excluding off duty time) to the 8-hour-day system. Even that time, with the lectures to attend and the necessary study, makes a busy and tiring day, leaving very little time for that recreation which is essential if we are to maintain a cheerful attitude to our patients.

Another point we would like to mention is the food. It is common knowledge that in most hospitals there is room for improvement, and also for a little variation of the menu. We would add that we like the work, and are desirous of obtaining our certificates; therefore we are willing to put up (or rather have to) with things as they are. All the same, we should like an alteration, but meanwhile prospective applicants knowing the conditions under which we live, are not encouraged to enter the profession.

Perhaps Mr. George Priestman will be interested to learn these facts. We cannot think that anyone taking up the profession because it is fashionable would be very successful. We hope that this letter will give you some idea why our girl-friends will not join us.

W. L. and A. J.

